# Capabilities of Human Biotissue Fluorescence Spectroscopy in the Wearable Multimodal Version

**DOI:** 10.17691/stm2025.17.3.03

**Published:** 2025-06-30

**Authors:** A.V. Dunaev, V.S. Yanushin, Yu.I. Loktionova, E.V. Zharkikh

**Affiliations:** Leading Researcher, Research and Development Center of Biomedical Photonics; Orel State University named after I.S. Turgenev, 95 Komsomolskaya St., Orel, 302026, Russia; Research Assistant, Research and Development Center of Biomedical Photonics; Master Student; Orel State University named after I.S. Turgenev, 95 Komsomolskaya St., Orel, 302026, Russia; Research Assistant, Research and Development Center of Biomedical Photonics; PhD Student; Orel State University named after I.S. Turgenev, 95 Komsomolskaya St., Orel, 302026, Russia; Researcher, Research and Development Center of Biomedical Photonics; Orel State University named after I.S. Turgenev, 95 Komsomolskaya St., Orel, 302026, Russia

**Keywords:** non-invasive optical diagnosis, fluorescence spectroscopy, biotissue fluorescence, skin fluorophores, microcirculatory- tissue system, oxidative metabolism of biotissues, multimodal approach, wearable analyzers, distributed system

## Abstract

Pathological processes in biotissues are characterized by a shift in metabolic homeostasis causing biochemical changes, which can be detected by fluorescence spectroscopy methods. A wide spectrum of capabilities, simple implementation, and valuable diagnostic information obtained attract considerable interest of the medical community.

**The aim of the study** is to analyze the current problems of fluorescence spectroscopy of biotissues and demonstrate new capabilities of this method in a wearable multimodal version for solving various problems of practical medicine.

**Theoretical part of the investigation:**

Factors influencing the registration of biotissue fluorescence have been considered. It has been established that the assessment of mitochondrial function (oxidative metabolism) by NADH and FAD fluorescence spectra is possible only under certain assumptions due to the difficulties in determining the contribution of collagen and some other fluorophores to the total spectrum. The capabilities of multimodal approach have been studied, i.e. сombining fluorescence spectroscopy and laser Doppler flowmetry in one diagnostic system as a wearable version of device implementation.

**Experimental part of the investigation:**

To demonstrate the capabilities of the wearable analyzers of the oxidative biotissue metabolism, pilot experimental investigations have been carried out involving 8 conditionally healthy volunteers. Parameters of microcirculatory-tissue systems (oxidative metabolism) were recorded with a modified multimodal wearable analyzer capable of measuring the skin fluorescence spectra in a wide range from 320 to 900 nm. Skin fluorescence was registered in the region of forehead, dorsal carpal surface, the volar surface of the distal phalanx of the middle finger, and the plantar surface of the distal phalanx of the first toe at a 365 nm wavelength of exciting irradiation.

The conducted experiment has shown that despite the existing effect of biotissue hyperemia together with the level of melanin on the recorded fluorescence spectrum, the assessment of skin fluorescence intensity in dynamics and functional tests reflect changes in metabolic processes of biotissues and may be considered as a promising diagnostic criterion.

## Introduction

In recent decades, methods of fluorescence spectroscopy (FS) and imaging become increasingly demanded for the diagnosis of metabolic processes in biological tissues [1]. FS is based on probing biotissues with optical radiation in ultraviolet or visible range with the following recording of autofluorescence spectra of endogenous and exogenous tissue fluorophores [2, 3]. Biological tissues are multicomponent structures with a complex chemical composition and contain a large variety of natural endogenous fluorophores characterized by different regions of absorption and emission, various quantum yields, and fluorescence lifetime. A general autofluorescence signal depends both on the quantitative content of fluorophores in the biological tissue and on their spatial distribution, and is also closely connected with the metabolic status of the biotissue and its morphology in normal or pathological conditions. Such coenzymes as reduced nicotinamide adenine dinucleotide (NADH) and flavin adenine dinucleotide (FAD), structural tissue proteins — collagen and elastin, amino acids — tryptophan and tyrosine, as well as porphyrins, lipofuscins, and melanin are referred to the substances possessing the most intensive autofluorescence in biological tissues [4–6]. In clinical practice, FS is used to diagnose pathological changes in biological tissues analyzing the difference in the parameters of intensity, lifetime, and spectral composition of fluorescence signal from normal and affected tissues [7, 8]. The fluorescence methods make it possible to detect at early stages the biochemical changes in tissues caused by pathological metabolic restructuring. Among the listed fluorophores, NADH and FAD are directly involved in the energy processes in the cells and are indicators of their metabolic status. Clinical studies have shown that variations of the parameters such as intensity and fluorescence lifetime of NADH and FAD caused by the changes in their concentration and interaction with different proteins reflect metabolic shifts induced by the pathological processes [7]. In this connection, monitoring of the biotissue fluorescence parameters may be employed to reveal oxidative metabolism. This approach is relevant for diagnosing diseases of various etiologies and for exploration of adaptation processes in the human body. Monitoring of fluorescence parameters is believed to be especially perspective in functional diagnosis, aerospace, and sports medicine [1, 9, 10].

Optical methods were applied for the first time in the 1950s to determine parameters of the respiratory chain and evaluate the mitochondrial function *in vitro* [11]. In 1965, a work was published, which demonstrated the capabilities of measuring NADH fluorescence for tracing the dynamics of metabolic activity in biological tissues *in vivo* [12]. In the next decades, FS has proved to be a promising method for early diagnosis of pathological processes by monitoring oxidative metabolism in biotissues [10]. In 1980–1990, there appeared the results of first investigations of using FS for differentiation of benign and malignant tumors of the breast and lungs [5, 13, 14]. By the present time, considerable progress has been achieved in employing fluorescence for revealing tumors of various organs [6, 15–17] and assessing metabolic activity of tissues in inflammatory processes [10, 18]. The first studies on employing the FS technique in the diagnosis and treatment covered gastroenterology, urology, gynecology [19–22]. Of special interest are investigations focused on the diagnosis and photodynamic therapy of oncological diseases [23, 24]. In a number of works, FS is used as a basic diagnostic technique together with a set of devices with thin-needle fiber-optic probes of small diameter to recognize tumors of the lungs [25, 26] and breast [27, 28]. In the last decades, alterations in the concentration of NADH, FAD and collagen in the tissues serves as a biomarker of tumor processes [29–31] and is used for determining the content of glycation end products in the tissues. It is especially important for patients with diabetes mellitus [32, 33], evaluation of skin condition in dermatology [34], and for other fields of biomedical research [35]. Thus, it is quite evident that FS has found a wide application in various fields of medicine, including oncology, transplantology, cosmetology, and surgery [36–40].

Nanoparticles labeled with fluorescent dyes are also used for blood flow analysis [41]. This method has some limitations connected, for example, with phototoxicity of ultraviolet radiation used in FS for fluorescence excitation. Safety of measurements in living tissues is ensured by limiting the radiant power density and duration of diagnostic procedures (for example, by pulsed modes of the fluorescence excitation sources).

**The aim of the study** is to analyze the current problems of using fluorescence spectroscopy of biotissues and demonstrate new capabilities of this technique in a wearable multimodal version for solving various problems of practical medicine.

## Theoretical part of the investigation

### Analysis of factors influencing the biotissue fluorescence recording

**Figure 1.**
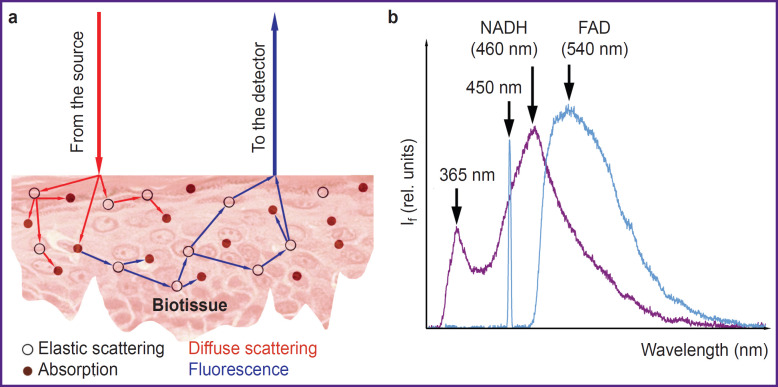
The principle of biotissue fluorescence spectroscopy implementation (a) and an example of recording fluorescence intensity spectra (I_f_) from the forearm skin for two wavelengths, 365 and 450 nm (b)

In the human skin there is a great number of natural fluorophores with close or overlapping spectral regions of absorption and fluorescence; therefore, fluorescent radiation emitted from the biotissue has a complex spectral composition. Multiple factors impact the recorded signal of biotissue fluorescence: temperature, optical properties of the examined specimen (scattering and absorption) [42], topological non-uniformity, technical characteristics of devices (the quality of excitation radiation sources, characteristics of photodetectors, specific features of the fiber optic end face, the distance between the source and receiver — the measurement base) [43], and others (Figure 1 (a)). Together, these factors directly influence the results of measurements, their dispersion and convergence, which determine the achievement of clinically significant and reliable results.

Currently, the following spectral ranges of skin fluorescence excitation are most commonly used to explore metabolic processes in biotissues: ultraviolet (350–380 nm) for recording NADH (maximal fluorescence in the range of 450–470 nm) and blue light for recording FAD (maximal fluorescence in the range 530–550 nm) [36]. An example of recording NADH and FAD fluorescence spectra from the skin of the healthy volunteer forearm for two excitation wavelengths (365 and 450 nm, respectively) is presented in Figure 1 (b).

It should be underlined that when using these wavelengths (365 and 450 nm) for excitation of skin fluorescence, a considerable contribution of collagen in the recorded spectrum should be taken into account (it is present in the dermis in the form of collagen fibers — 75% of dermal tissue) [44, 45]. Besides, in addition to the mentioned fluorophores (NADH and FAD), keratin, bilirubin, porphyrins, and carotenoids also make their contribution to the skin spectrum in the range of 400–480 nm, making the analysis of the recorded data more difficult. Biotissue blood filling also influences considerably the skin fluorescence spectra due to the absorption of optical radiation by hemoglobin: the intensity of fluorescence decreases with the increase of blood volume in the dermis [46]. Thus, the application of local pressure by optical probes, which are used to register the fluorescence, impacts biotissue optical characteristics, since it changes blood content in the superficial vascular plexus of the dermis. This factor should also be considered when conducting investigations. Besides, presence of melanin pigment in the epidermis has a strong effect on the skin fluorescence spectrum because of a high absorption of ultraviolet and visible light [47].

**Figure 2.**
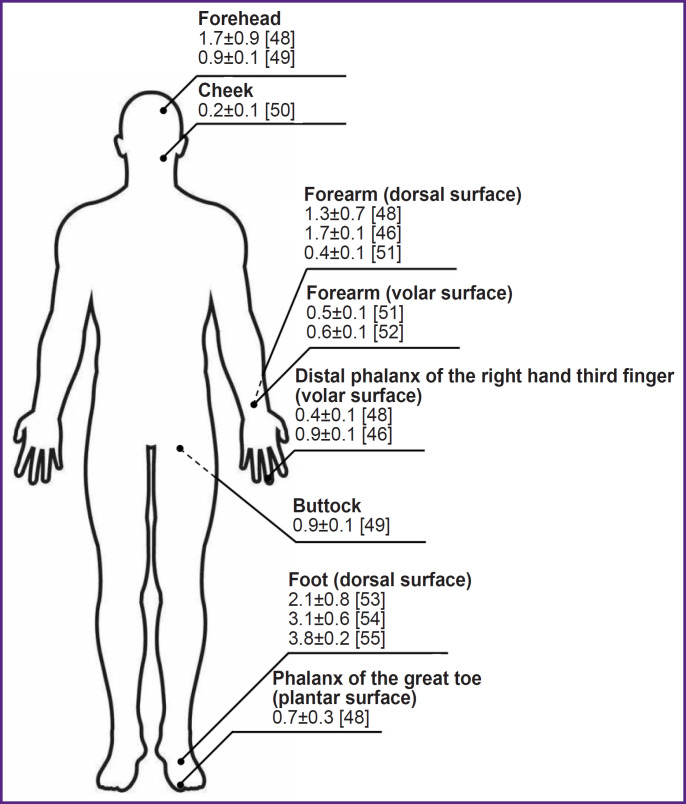
Examples of recording amplitudes of skin fluorescence intensity normalized to the back-scattered radiation at the 460 nm wavelength and excitation by 350–370 nm wavelengths (rel. units)

Normalizing intensity maxima to back-scattered radiation intensity values at the exciting wavelength is one of the approaches to take into account the effect of various factors during skin fluorescence recording (primarily, biotissue blood filling). Figure 2 shows examples of fluorescent intensity amplitudes (in relative units) for different human skin regions at 460 nm wavelength at the excitation by the 350–370 nm wavelengths, normalized to the back-scattered radiation [46, 48–55]. The analysis of the presented data demonstrates their high variability, which results from the effect of all factors mentioned above. On average, individual variability for the fluorescence intensity of the main biotissue fluorophores (for example, in the assessment of the results of measuring NADH and FAD, including the collagen effect) is about 30%, which should be considered when interpreting the data of the skin FS [56].

Thus, the evaluation of the mitochondrial function (oxidative metabolism) using the recorded NADH and FAD spectra is possible only with certain assumptions, since the effect of collagen and other factors on the biotissue fluorescence recording is still unsolved problem. Besides, to account for different levels of biotissue blood filling, it is necessary to normalize fluorescence intensity, for example, to the back-scattered radiation.

### Wearable implementation of biotissue fluorescence spectroscopy based on multimodal approach

In view of a number of factors limiting a wide introduction of FS in clinical practice, a new approach is proposed: to combine FS and laser Doppler flowmetry (LDF) in one diagnostic system [1].

Laser Doppler flowmetry is a widely spread method of non-invasive optic diagnosis used to evaluate functional condition of the microcirculatory part of the vascular bed. This method is based on probing the skin with coherent laser radiation with subsequent recording of the radiation intensity back-scattered from the static tissue structures and moving blood cells — erythrocytes. After the photometric intensity measurement of the back-scattered radiation and its analog-to-digital conversion, the index of microcirculation is calculated, which is proportional to the number of erythrocytes in the tested tissue volume and the mean rate of their movement. Information on the functioning of endothelial, neurogenic, myogenic, respiratory, and pulse mechanisms of blood flow regulation is contained in the obtained signal [1].

Combining LDF and FS in one diagnostic system allows one not only to assess concurrently the peripheral blood flow and metabolic activity of the cells but to calculate complex parameters (for example, oxidative metabolism index) [57, 58]. This indicator reflects efficiency and coordinated work of the systems delivering nutrients and oxygen to biological tissues and their consumption in oxidative metabolism. In a wider sense, this indicator helps obtain information on functional state of microcirculatory-tissue systems (MTS) of the human body [59]. Application of the oxidative metabolism index as one of the indicators of the organism homeostasis is a promising direction within the frames of personified medicine development.

The multimodal approach has attracted wide interest of the scientific community and is being actively implemented into clinical practice to improve the quality of early diagnosis of metabolic and perfusion impairment in various diseases, including socially significant disorders. Thus, data on the differences in the accumulation of glycation end products in biotissues of patients with type 2 diabetes mellitus relative to conditionally healthy volunteers were obtained in clinical conditions [55]. The method, based on the multimodal approach and using signal records from FS and LDF, makes it possible to classify the biotissue state in terms of the presence or absence of microcirculatory- metabolic disorders with the probability of obtaining a false-negative diagnostic result below 0.2 with subsequent differentiation of the pathology severity degree.

Until recently, application of the multimodal approach to MTS diagnosing was possible mainly in the conditions of clinical studies or research laboratories, due to the usage of stationary devices with fiber-optic probes requiring the appropriate operator qualification. An example of such device is LAKK-M diagnostic complex (SPE “LAZMA” Ltd., Russia). The presence of a fiberoptic probe is an essential factor influencing the recorded signal. Difficulty of its mounting and fixation on the patient body as well as high sensitivity to motion artefacts complicate considerably the process of data registration for both operators and examined individuals.

Owing to the development of technologies for the production of optic components, it became possible to create compact radiation sources and spectrometers. This, for example, has led to the appearance of the wearable multimodal LAZMA PF analyzer (SPE “LAZMA” Ltd., Russia), which considerably widened the spectrum of diagnostic capabilities relative to the stationary modifications. These kinds of devices may be fixed on symmetrical or random skin regions, creating a distributed system of devices on the human body. These analyzers implement LDF and FS methods and provide registration of the skin surface temperature and motion artifacts using accelerometer. Data are transmitted through wireless communication channels (Bluetooth or Wi-Fi). FS channel in the portable version is implemented using the light-emitting diode (LED) with the 365 nm excitation wavelength operating in the pulsed mode. The spectrometer serves as a receiver, allowing one to obtain spectral distribution of human biotissue fluorescence. The LED wavelength was selected based on the spectral excitation characteristic of the NADH coenzyme.

Simultaneous implementation of LDF and FS in LAZMA PF system allows for the complex assessment of MTS taking into account metabolic activity of the biological tissue. Wearable devices are gradually finding wide application in the diagnosis of complications related to diabetes mellitus [60], hypertension [61], and vascular complications of coronavirus infection [62]. The LAZMA PF analyzers have been tried and tested in the evaluation of smoking status [63], drug therapy of blood microcirculation disorders [64], and the quality of performing breathing exercises [65].

Therefore, the multimodal approach, including several methods of non-invasive optical diagnosis, and implemented in the wearable version, gives the opportunity to synthesize novel diagnostic criteria for early detection of socially significant diseases, which speaks of a high potential of applying wearable devices in various fields of medicine, including telemedicine.

## Experimental part of the investigation

To demonstrate the possibilities and specific features of application of the proposed wearable analyzers, pilot experimental investigations involving 8 conditionally healthy volunteers aged from 18 to 23 years (6 men, 2 women) have been carried out. All participants did not have any diagnosed chronic diseases and did not take medicines on a regular basis. Presence of harmful habits was the criterion for exclusion from the study.

The study complies with the Declaration of Helsinki (2024) and was approved by the Ethical Committee of Orel State University named after I.S. Turgenev. Written informed consent was obtained from every participant.

To study the MTS parameters, we used a specially fabricated modification of the wearable LAZMA PF analyzers, which allowed recording the spectra of skin fluorescence in a wide range of 320–900 nm. This modification gives the opportunity to assess spectral distribution of the excited fluorophore, the level of fluorescence intensity, and the intensity of the back- scattered radiation at the source wavelength. Owing to this, individual morphological features of the biotissue can be taken into account by normalizing the fluorescent signal.

The analyzer was alternately fixed on the forehead skin (which is supplied with blood by the supraorbital artery from the basin of the internal carotid artery) and on the plantar surface of the distal phalanges of the great toes (a region with a great number of arteriolo-venular anastomoses). During measurements, the volunteers were sitting at the table with a straight back and hands located on the table at the level of the heart.

To study the effect of the biotissue blood filling level on the spectrum of skin fluorescence, a series of measurements of fluorescence intensity in the skin of the fingers and forearms was done. The study protocol included simultaneous recording of LDF and FS signals and 3 stages of the occlusion test (reactive hyperemia test): the basic recording of the MTS parameters was performed for 7 min; next, arterial occlusion was induced by pressing the arm artery for 3 min with a tonometer pressure cuff, which was inflated until pressure of about 200 mm Hg was reached; after removing the occlusion and post-occlusion reactive hyperemia, restoration of the MTS parameters were recorded for 8 min.

All measurements were performed in normal conditions at 22±1°С room temperature and at least 2 h after food intake. The volunteers were in a calm state and has been adapting to the environment for 20–30 min before the beginning of measurements.

**Figure 3.**
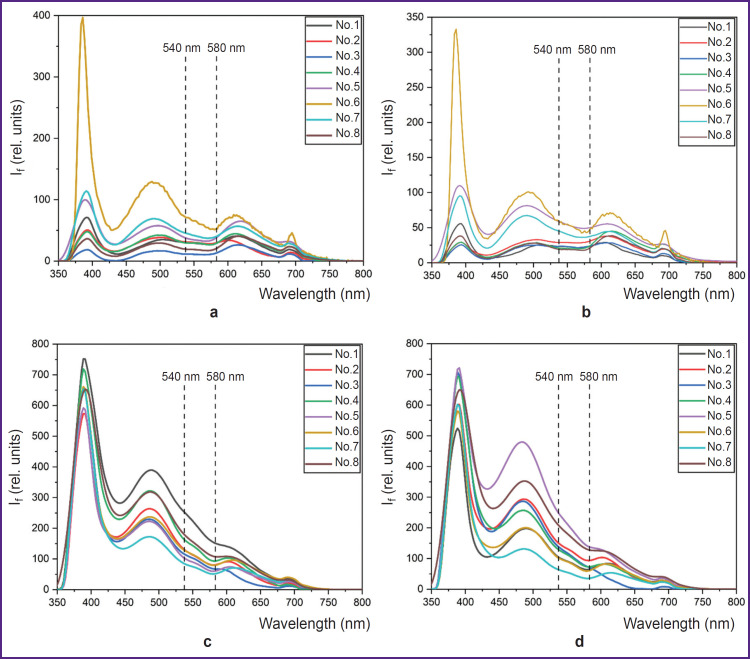
Fluorescence intensity spectra (I_f_) of the skin in various regions: (a) forehead on the right; (b) forehead on the left; (c) plantar surface of the distal phalanx of the right foot great toe; (d) plantar surface of the distal phalanx of the left foot great toe

Spectra of fluorescence in the region of the forehead skin (a), (b) and toes (c), (d) obtained for 8 conditionally healthy volunteers are presented in Figure 3. At excitation by 365 nm wavelength, a characteristic peak of NADH fluorescence is observed at 460 nm wavelength.

Volunteers’ fluorescence spectrum shapes in each tested region are identical, however, there are some differences in the spectrum shapes in various regions of measurement. The fluorescence spectrum of volunteer No.6 in the region of the forehead skin is characterized by the largest amplitude of the back-scattered radiation and NADH fluorescence, which is most likely associated with a lighter skin, i.e. with a smaller content of melanin in epidermis. High values of fluorescence intensity variability may also be explained by different melanin content of the participants. To decrease the dispersion, it is reasonable to normalize intensity values to the back- scattered radiation. This will take into account individual optical properties of participants’ skin.

It is worth mentioning that in the region of the plantar surface of the great toe distal phalanges, variability of fluorescence intensity amplitude at 460 nm wavelength was 42 and 25% on the left and right, respectively. Decrease of variability may be connected with the fact that melanin impacts the fluorescence spectrum to lesser extent, since this region is actually not exposed to ultraviolet radiation. The differences in the shape and amplitudes of fluorescence spectra between the men and women were not found.

**Figure 4.**
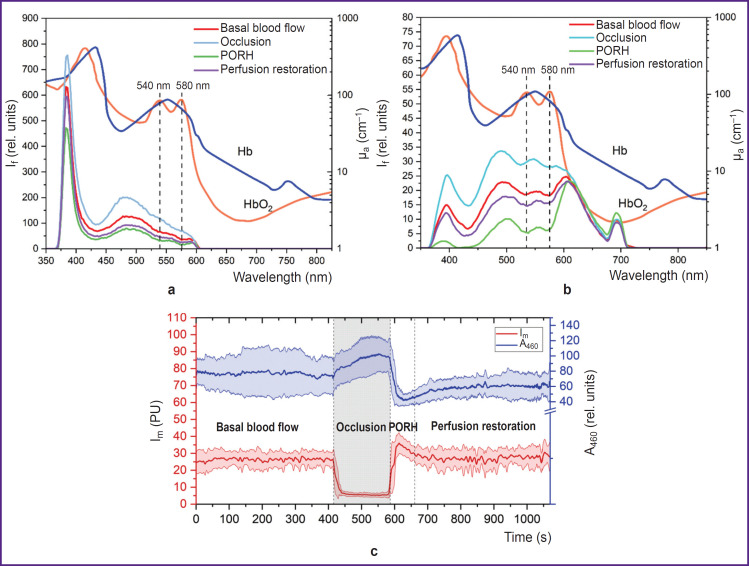
Examples of skin fluorescence intensity spectra (I_f_) with the curves of hemoglobin and oxyhemoglobin absorption (a), (b) and dynamics of changes in the parameters of laser Doppler flowmetry and fluorescence spectroscopy (c) during the occlusion test in different measured regions: (a) volar surface of the right hand third finger; (b) dorsal surface of the forearm; (c) fluorescence intensity at 460 nm (A_460_) with the record of index of microcirculation (I_m_) in the region of the volar surface of the distal phalanx of the right hand third finger; μ_a_ — transport absorption coefficient; PORH — post-occlusion reactive hyperemia; HbO_2_ — oxyhemoglobin, Hb — hemoglobin

The data analysis has confirmed that the fluorescence spectrum is greatly influenced by blood filling of the biological tissues in the form of a characteristic signal decrease at about 540 and 580 nm wavelengths corresponding to the absorption of the radiation by oxyhemoglobin (HbO_2_), which should also be considered when interpreting the obtained results. The skin fluorescence spectra for different stages of the occlusion test are presented in Figure 4 (a), (b). Elevation of fluorescence intensity at the stage of occlusion and its reduction in post-occlusion reactive hyperemia also validates a considerable impact of tissue blood filling on the recorded signal. Lower peak intensity of the back- scattered radiation in the region of dorsal wrist surface may be caused by high radiation absorption due to the increased content of melanin and angioarchitectonics of the tested region.

Figure 4 (c) illustrates the dynamics of changes in the index of microcirculation and fluorescence intensity at 460 nm wavelength during the occlusion test. Despite the evident inverse dependence of fluorescence intensity on blood filling, the effect of other factors is also observed. For example, after removing blood flow occlusion, restoration of tissue reperfusion occurs up to the base values, although the fluorescence intensity remains at a lower level relative to the initial values. This probably reflects compensatory changes of oxidative metabolism in response to hypoxia seen as a reduction of quantitative ratio of NADH in the biotissue cells. Besides, it is worth noting that after the occlusion test, fluorescence intensity dispersion decreases in comparison with the period prior to occlusion, which may be the result of triggering a cascade of adaptation reactions in response to the irritating effect.

Thus, the conducted experiment has shown that despite the considerable impact of the biotissue hyperemia together with melanin on the recorded fluorescence spectrum at the 365 nm excitation wavelength, the assessment of the skin fluorescence intensity in dynamics by functional tests reflects the changes of metabolic processes in biological tissues and is a promising diagnostic criterion.

## Conclusion

Fluorescence spectroscopy of biotissues is a promising non-invasive technique allowing for objective assessment of functional condition of human biological tissues. Fluorescence of endogenous fluorophores may be an effective diagnostic criterion for determination of metabolic activity of the cellular respiratory chain, owing to which it is possible to detect neoplasms, including malignant ones.

Application of the multimodal approach, which consists in the simultaneous implementation of fluorescence spectroscopy with other methods of biophotonics, allows one to receive complex information on the efficiency and consistency of the processes of oxygen and nutrient delivery by the blood system and their utilization by the biological tissues, which widens considerably the diagnostic capabilities. New formats of fluorescence spectroscopy realization in the form of integration in the distributed system of portable devices open novel perspectives of using this technique in the field of functional diagnosis to improve the quality of personified medicine.

## References

[1] Dunaev A.V. (2022). Mul’timodal’naya opticheskaya diagnostika mikrotsirkulyatorno-tkanevykh sistem organizma cheloveka [Multimodal optical diagnostics of microcirculatory tissue systems of the human body]..

[2] Rogatkin D.A. (2014). Physical foundations of laser clinical fluorescence spectroscopy in vivo.. Meditsinskaya fizika.

[3] Zherebtsov E.A., Dremin V.V., Zherebtsova A.I., Potapova E.V., Dunaev A.V. (2008). Fluorestsentnaya diagnostika mitokhondrial’noy funktsii v epitelial’nykh tkanyakh in vivo [Fluorescence diagnostics of mitochondrial function in epithelial tissues in vivo]..

[4] Richards-Kortum R., Sevick-Muraca E. (1996). Quantitative optical spectroscopy for tissue diagnosis.. Annu Rev Phys Chem.

[5] Alfano R., Tata D.B., Cordero J., Tomashefsky P., Longo F., Alfano M. (1984). Laser induced fluorescence spectroscopy from native cancerous and normal tissue.. IEEE J Quantum Electron.

[6] Georgakoudi I., Jacobson B.C., Müller M.G., Sheets E.E., Badizadegan K., Carr-Locke D.L., Crum C.P., Boone C.W., Dasari R.R., Van Dam J., Feld M.S. (2002). NAD(P)H and collagen as in vivo quantitative fluorescent biomarkers of epithelial precancerous changes.. Cancer Res.

[7] Koenig K., Schneckenburger H. (1994). Laser-induced autofluorescence for medical diagnosis.. J Fluoresc.

[8] Croce A.C., Bottiroli G. (2014). Autofluorescence spectroscopy and imaging: a tool for biomedical research and diagnosis.. Eur J Histochem.

[9] Mayevsky A., Chance B. (2007). Oxidation-reduction states of NADH in vivo: from animals to clinical use.. Mitochondrion.

[10] Mayevsky A. (2015). Mitochondrial function in vivo evaluated by NADH fluorescence.. Springer;..

[11] Chance B., Williams G.R. (1955). Respiratory enzymes in oxidative phosphorylation. I. Kinetics of oxygen utilization.. J Biol Chem.

[12] Chance B., Williamson J.R., Jamieson D., Schoener B. (1965). Properties and kinetics of reduced pyridine nucleotide fluorescence of the isolated and in vivo rat heart.. Biochem Zeit.

[13] Alfano R.R., Das B.B., Cleary J., Prudente R., Celmer E.J. (1991). Light sheds light on cancer — distinguishing malignant tumors from benign tissues and tumors.. Bull N Y Acad Med.

[14] Alfano R., Tang G.C., Pradhan A., Lam W., Choy D., Opher E. (1987). Fluorescence spectra from cancerous and normal human breast and lung tissues.. IEEE J Quantum Electron.

[15] Palmer G.M., Keely P.J., Breslin T.M., Ramanujam N. (2003). Autofluorescence spectroscopy of normal and malignant human breast cell lines.. Photochem Photobiol.

[16] Palmer S., Litvinova K., Dunaev A., Yubo J., McGloin D., Nabi G. (2017). Optical redox ratio and endogenous porphyrins in the detection of urinary bladder cancer: a patient biopsy analysis.. J Biophotonics.

[17] Panjehpour M., Julius C.E., Phan M.N., Vo-Dinh T., Overholt S. (2002). Laser-induced fluorescence spectroscopy for in vivo diagnosis of non-melanoma skin cancers.. Lasers Surg Med.

[18] Croce A.C., Bottiroli G. (2014). Autofluorescence spectroscopy and imaging: a tool for biomedical research and diagnosis.. Eur J Histochem.

[19] Apolikhina L.A., Denisova E.D., Bulgakova N.N., Kuzmin S.G., Vorozhtsov G.N. (2006). Fluorescence detection and photodynamic therapy of human papilloma virus associated diseases of female genital organs.. Proceedings of the 6th International Congress of the World Association of Laser Therapy, WALT 2006..

[20] Bulgakova N., Sokolov V., Telegina L., Vereshchagin K., Frank G., Chissov V. (2013). Study of laser- induced autofluorescence emission spectra from normal and malignant bronchial epithelium.. Photonics Lasers Med.

[21] Rusakov I.G., Sokolov V.V., Bulgakova N.N., Ul’ianov R.V., Teplov A.A. (2008). Fluorescent diagnostic methods and superficial cancer of the urinary bladder: current status.. Urologiia.

[22] Dronova O.B., Tretyakov A.A., Mishchenko A.N., Bulgakova N.N. (2008). Laser induced autofluorescence of normal and metaplastic epithelium of esophagogastric transition at gastroesophageal reflux disease.. Sibirskiy onkologicheskiy zhurnal.

[23] Bulgakova N.N., Kazachkina N.I., Sokolov V.V., Smirnov V.V. (2006). Local fluorescence spectroscopy and detection of malignancies using laser excitation at various wavelengths.. Laser Phys.

[24] Bulgakova N., Ulijanov R., Vereschagin K., Sokolov V., Teplov A., Rusakov I., Chissov V. (2009). In vivo local fluorescence spectroscopy in PDD of superficial bladder cancer.. Medical Laser Application.

[25] Harris K., Rohrbach D.J., Attwood K., Qiu J., Sunar U. (2017). Optical imaging of tissue obtained by transbronchial biopsies of peripheral lung lesions.. J Thorac Dis.

[26] Braun F., Schalk R., Nachtmann M., Hien A., Frank R., Beuermann T., Methner F.J., Kränzlin B., Rädle M., Gretz N. (2019). A customized multispectral needle probe combined with a virtual photometric setup for in vivo detection of Lewis lung carcinoma in an animal model.. Meas Sci Technol.

[27] Mathieu M.C., Toullec A., Benoit C., Berry R., Validire P., Beaumel P., Vincent Y., Maroun P., Vielh P., Alchab L., Farcy R., Moniz-Koum H., Fontaine-Aupart M.P., Delaloge S., Balleyguier C. (2018). Preclinical ex vivo evaluation of the diagnostic performance of a new device for in situ label-free fluorescence spectral analysis of breast masses.. Eur Radiol.

[28] Spliethoff J.W., Evers D.J., Jaspers J.E., Hendriks B.H., Rottenberg S., Ruers T.J. (2014). Monitoring of tumor response to Cisplatin using optical spectroscopy.. Transl Oncol.

[29] Ostrander J.H., McMahon C.M., Lem S., Millon S.R., Brown J.Q., Seewaldt V.L., Ramanujam N. (2010). Optical redox ratio differentiates breast cancer cell lines based on estrogen receptor status.. Cancer Res.

[30] Sivabalan S., Vedeswari C.P., Jayachandran S., Koteeswaran D., Pravda C., Aruna P.R., Ganesan S. (2010). In vivo native fluorescence spectroscopy and nicotinamide adinine dinucleotide/flavin adenine dinucleotide reduction and oxidation states of oral submucous fibrosis for chemopreventive drug monitoring.. J Biomed Opt.

[31] Palmer S., Litvinova K., Rafailov E.U., Nabi G. (2015). Detection of urinary bladder cancer cells using redox ratio and double excitation wavelengths autofluorescence.. Biomed Opt Express.

[32] Fokkens B.T., Smit A.J. (2016). Skin fluorescence as a clinical tool for non-invasive assessment of advanced glycation and long-term complications of diabetes.. Glycoconj J.

[33] Bos D.C., de Ranitz-Greven W.L., de Valk H.W. (2011). Advanced glycation end products, measured as skin autofluorescence and diabetes complications: a systematic review.. Diabetes Technol Ther.

[34] Galkina E.M., Utts S.R. (2013). Fluorescent diagnostics in dermatology.. Saratovskiy nauchno-meditsinskiy zhurnal.

[35] Kang U.K., Papayan G.V., Berezin V.B., Petrishchev N.N., Galagudza M.M. (2013). Spectrometer for fluorescence-reflectance biomedical research.. Opticheskiy zhurnal.

[36] Tuchin V. (2007). Opticheskaya biomeditsinskaya diagnostika. V 2 t. T. 2 [Optical biomedical diagnostics. In 2 vol. Vol. 2]..

[37] Castro-e-Silva O., Sankarankutty A.K., Correa R.B., Ferreira J., Vollet Filho J.D., Kurachi C., Bagnato V.S. (2008). Autofluorescence spectroscopy in liver transplantation: preliminary results from a pilot clinical study.. Transplant Proc.

[38] Ershova E.Y., Karimova L.N., Kharnas S.S., Kuzmin S.G., Loschenov V.B. (2003). Photodynamic therapy of acne vulgaris.. Lasers in surgery: advanced characterization, therapeutics, and systems XIII..

[39] De Veld D.C., Witjes M.J., Sterenborg H.J., Roodenburg J.L. (2005). The status of in vivo autofluorescence spectroscopy and imaging for oral oncology.. Oral Oncol.

[40] Akbar N., Sokolovski S., Dunaev A., Belch J.J., Rafailov E., Khan F. (2014). In vivo noninvasive measurement of skin autofluorescence biomarkers relate to cardiovascular disease in mice.. J Microsc.

[41] Tarakanchikova Y., Stelmashchuk O., Seryogina E., Piavchenko G., Zherebtsov E., Dunaev A., Popov A., Meglinski I. (2018). Allocation of rhodamine-loaded nanocapsules from blood circulatory system to adjacent tissues assessed in vivo by fluorescence spectroscopy.. Laser Phys Lett.

[42] Lin Y., Gao H., Nalcioglu O., Gulsen G. (2007). Fluorescence diffuse optical tomography with functional and anatomical a priori information: feasibility study.. Phys Med Biol.

[43] Rogatkin D.A., Prisnyakova O.A., Moiseeva L.G., Cherkasov A.S. (1998). Analysis of the accuracy of clinical laser fluorescence diagnosis.. Izmeritel’naya tekhnika.

[44] Sinichkin Y.P., Utz S.R., Mavliutov A.H., Pilipenko H.A. (1998). In vivo fluorescence spectroscopy of the human skin: experiments and models.. J Biomed Opt.

[45] Konig K., Riemann I. (2003). High-resolution multiphoton tomography of human skin with subcellular spatial resolution and picosecond time resolution.. J Biomed Opt.

[46] Dunaev A.V., Dremin V.V., Zherebtsov E.A., Rafailov I.E., Litvinova K.S., Palmer S.G., Stewart N.A., Sokolovski S.G., Rafailov E.U. (2015). Individual variability analysis of fluorescence parameters measured in skin with different levels of nutritive blood flow.. Med Eng Phys.

[47] Dremin V.V., Dunaev A.V. (2016). How the melanin concentration in the skin affects the fluorescence-spectroscopy signal formation.. J Opt Technol.

[48] Parshakova V.E., Zharkikh E.V., Loktionova Yu.I., Koskin A.V., Dunaev A.V. (2024). Study of physiologic variabity of microcirculatory-tissue systems parameters of human organism using multimodal portable analyzers.. Fundamental’nye i prikladnye problemy tekhniki i tekhnologii.

[49] Na R., Stender I.M., Ma L., Wulf H.C. (2000). Autofluorescence spectrum of skin: component bands and body site variations.. Skin Res Technol.

[50] Kollias N., Zonios G., Stamatas G.N. (2002). Fluorescence spectroscopy of skin.. Vib Spectrosc.

[51] Ryzhkova E., Morgunova T., Potapova E., Ryzhkov I., Fadeyev V. (2024). Fluorescence spectroscopy with temperature functional tests in the assessment of markers of intracellular energy metabolism: spatial heterogeneity and reproducibility of measurements.. J Biophotonics.

[52] Gillies R., Zonios G., Anderson R.R., Kollias N. (2000). Fluorescence excitation spectroscopy provides information about human skin in vivo.. J Invest Dermatol.

[53] Potapova E.V., Dremin V.V., Zherebtsov E.A., Makovik I.N., Zharkikh E.V., Dunaev A.V., Pilipenko O.V., Sidorov V.V., Krupatkin A.I. (2017). A complex approach to noninvasive estimation of microcirculatory tissue impairments in feet of patients with diabetes mellitus using spectroscopy.. Optics and Spectroscopy.

[54] Dremin V.V. (2016). Analytical review of approaches to mathematical modeling of biological tissue fluorescence.. Fundamental’nye i prikladnye problemy tekhniki i tekhnologii.

[55] Dremin V.V., Zherebtsov E.A., Sidorov V.V., Krupatkin A.I., Makovik I.N., Zherebtsova A.I., Zharkikh E.V., Potapova E.V., Dunaev A.V., Doronin A.A., Bykov A.V., Rafailov I.E., Litvinova K.S., Sokolovski S.G., Rafailov E.U. (2017). Multimodal optical measurement for study of lower limb tissue viability in patients with diabetes mellitus.. J Biomed Opt.

[56] Dunajev A.V., Drjomin V.V., Gherebtsov E.A., Palmer S.G., Sokolowskiy S.G., Rafailov E.U. (2013). Analisys of individual variability of parameters of laser fluorescence diagnostics.. Biotekhnosfera.

[57] Dunaev A. (2023). Wearable devices for multimodal optical diagnostics of microcirculatory-tissue systems: application experience in the clinic and space.. J Biomed Photonics Eng.

[58] Sidorov V.V., Rybakov Y.L., Gukasov V.M., Evtushenko G.S. (2022). A system of local analyzers for noninvasive diagnostics of the general state of the tissue microcirculation system of human skin.. Biomed Eng (NY).

[59] Dunaev A.V. (2020). Principles of construction of technical means multiparametric optical diagnostics for assessing the functional state of microcirculatory-tissue systems.. Fundamental’nye i prikladnye problemy tekhniki i tekhnologii.

[60] Loktionova Yu.I., Zharkikh E.V., Zherebtsova A.I., Kozlov I.O., Zherebtsov E.A., Masalygina G.I., Dunaev A.V. (2019). Study of age-related and pathological features of microhemodynamics in healthy volunteers and patients with type 2 diabetes mellitus by wearable laser Doppler flowmetry devices.. Fundamental’nye i prikladnye problemy tekhniki i tekhnologii.

[61] Gorshkov A.Yu., Korolev A.I., Fedorovich A.A., Omelyanenko K.V., Dadaeva V.A., Drapkina O.M. (2022). Parameters of skin perfusion according to the data of remote laser doppler flowmetry in men with newly detected arterial hypertension.. Profilakticheskaya meditsina.

[62] Fedorovich A.A., Markov D.S., Malishevsky M.V., Yudakov O.O., Gorshkov A.Yu., Baldin A.V., Zhuk D.M., Spasenov A.Yu., Korolev A.I., Koptelov A.V., Drapkina O.M. (2022). Microcirculatory disorders in the forearm skin in the acute phase of COVID-19 according to laser Doppler flowmetry.. Regional blood circulation and microcirculation.

[63] Saha M., Dremin V., Rafailov I., Dunaev A., Sokolovski S., Rafailov E. (2020). Wearable laser doppler flowmetry sensor: a feasibility study with smoker and non-smoker volunteers.. Biosensors (Basel).

[64] Zharkikh E.V., Loktionova Yu.I., Sidorov V.V., Krupatkin A.I., Masalygina G.I., Dunaev A.V. (2022). Control of blood microcirculation parameters in therapy with alpha- lipoic acid in patients with diabetes mellitus.. Human Physiology.

[65] Frolov A.V., Loktionova Yu.I., Zharkikh E.V., Sidorov V.V., Krupatkin A.I., Dunaev A.V. (2021). Investigation of changes in the skin blood microcirculation when performing the hatha yoga breathing technique.. Regional blood circulation and microcirculation.

